# Developing EMR-based algorithms to Identify hospital adverse events for health system performance evaluation and improvement: Study protocol

**DOI:** 10.1371/journal.pone.0275250

**Published:** 2022-10-05

**Authors:** Guosong Wu, Cathy Eastwood, Yong Zeng, Hude Quan, Quan Long, Zilong Zhang, William A. Ghali, Jeffrey Bakal, Bastien Boussat, Ward Flemons, Alan Forster, Danielle A. Southern, Søren Knudsen, Brittany Popowich, Yuan Xu

**Affiliations:** 1 Centre for Health Informatics, Department of Community Health Sciences, Cumming School of Medicine, University of Calgary, Calgary, Alberta, Canada; 2 Concordia Institute for Information Systems Engineering, Gina Cody School of Engineering and Computer Science, Concordia University, Montreal, Quebec, Canada; 3 Department of Biochemistry and Molecular Biology, Department of Medical Genetics, Department of Mathematics and Statistics, University of Calgary, Calgary, Alberta, Canada; 4 Alberta Children’s Hospital Research Institute, Calgary, Alberta, Canada; 5 Hotchkiss Brain Institute, Calgary, Alberta, Canada; 6 Office of Vice President of Research & O’Brien Institute of Public Health, University of Calgary, Calgary, Alberta, Canada; 7 Provincial Research Data Services, Data and Analytics, Alberta Health Services, Calgary, Alberta, Canada; 8 Alberta Health Services, Calgary, Alberta, Canada; 9 Clinical Epidemiology and Quality of Care Unit, University Grenoble Alpes, Faculty of Medicine, Grenoble University Hospital, France; 10 Department of Medicine, Cumming School of Medicine, University of Calgary, Calgary, Alberta, Canada; 11 Department of Clinical Epidemiology, Ottawa Hospital Research Institute, Ottawa, Ontario, Canada; 12 Digital Design Department, IT University of Copenhagen, Copenhagen, Denmark; 13 Department of Oncology, Cumming School of Medicine, University of Calgary, Calgary, Alberta, Canada; 14 Department of Surgery, Cumming School of Medicine, University of Calgary, Calgary, Alberta, Canada; GERMANY

## Abstract

**Background:**

Measurement of care quality and safety mainly relies on abstracted administrative data. However, it is well studied that administrative data-based adverse event (AE) detection methods are suboptimal due to lack of clinical information. Electronic medical records (EMR) have been widely implemented and contain detailed and comprehensive information regarding all aspects of patient care, offering a valuable complement to administrative data. Harnessing the rich clinical data in EMRs offers a unique opportunity to improve detection, identify possible risk factors of AE and enhance surveillance. However, the methodological tools for detection of AEs within EMR need to be developed and validated. The objectives of this study are to develop EMR-based AE algorithms from hospital EMR data and assess AE algorithm’s validity in Canadian EMR data.

**Methods:**

Patient EMR structured and text data from acute care hospitals in Calgary, Alberta, Canada will be linked with discharge abstract data (DAD) between 2010 and 2020 (n~1.5 million). ***AE algorithms development***. First, a comprehensive list of AEs will be generated through a systematic literature review and expert recommendations. Second, these AEs will be mapped to EMR free texts using Natural Language Processing (NLP) technologies. Finally, an expert panel will assess the clinical relevance of the developed NLP algorithms. ***AE algorithms validation***: We will test the newly developed AE algorithms on 10,000 randomly selected EMRs between 2010 to 2020 from Calgary, Alberta. Trained reviewers will review the selected 10,000 EMR charts to identify AEs that had occurred during hospitalization. Performance indicators (e.g., sensitivity, specificity, positive predictive value, negative predictive value, F_1_ score, etc.) of the developed AE algorithms will be assessed using chart review data as the reference standard.

**Discussion:**

The results of this project can be widely implemented in EMR based healthcare system to accurately and timely detect in-hospital AEs.

## Background

Several data collection approaches have been developed for patient safety surveillance globally, including voluntary hospital reports of nosocomial infections, a nationally representative survey of drug-drug interactions, and regional and national voluntary reporting of adverse events (AEs) and medical errors. For example, Baker et al. reviewed 3,745 charts to record the presence of 17 AEs in five Canadian provinces [1]. They reported an AE rate of 7.5 per 100 hospital admissions. Among AEs detected in that work, 37% were considered preventable, and 20% were fatal. However, these data collection methods focus on a specific type of event, collect data from non-random and biased populations, cover limited geographic areas, or are too labor-intensive for widespread use [2]. Therefore, researchers have paid great attention to routinely collected administrative data for population-based studies of AEs.

Recognition of these primary data collection issues led to the Agency for Healthcare Research and Quality (AHRQ) and the U of California-Stanford Evidence-based Practice Centre, to conduct pioneering work to develop 20 hospital Patient Safety Indicators (PSIs) for use with ICD-9-CM administrative data [3]. These PSIs are readily available and relatively inexpensive to use [3]. The PSIs can help hospitals identify potential AEs that need further study and provide an opportunity to assess the occurrence of AEs and in-hospital complications using routinely collected administrative data. However, the validity of PSIs in ICD-10 administrative data is problematic. While the codes accurately define an event, the clinical vagueness inherent in the description of the code itself (*e*.*g*., “hypotension”) may lead to a highly heterogeneous pool of clinical states represented by that code. In prior work, we assessed if the AHRQ PSIs could be used for case finding in Canadian administrative health data through chart reviews [2]. Of the five PSIs reviewed, the positive predictive value (PPV) for laceration was 86.4%, pulmonary embolism and deep vein thrombosis was 89.5%, infection was 79.1%, foreign body due to surgery was 62.5%, and sepsis was 12.5% [2].

Electronic Medical Records (EMRs) are systemized collections of patient health information and documentation, collected in real-time and stored in digital format. EMRs were initially designed to facilitate clinical decision-making regarding health care delivery for individual patients and improve care quality. Over the past decade, EMRs have seen rapid deployment in health care worldwide. The adoption rate has differed by provinces in Canada [4, 5]. In Calgary, a city-wide inpatient EMR system called AllScripts Sunrise Clinical Manager (SCM) has been operating since 2006. Since its inception, SCM EMR has collected longitudinal hospital health data on 5,469,761 individuals [6]. Since 2019, Alberta Health Services has implemented Connect Care, which offers a province-wide EMR system. Huge amount of abundant clinical information documented in EMR provides both challenges and opportunities. There is a growing need to understand EMR data, ultimately allowing researchers to leverage the data to optimize patient care.

With the widespread implementation of EMRs, data science methodologies have been developed for mining these rich unstructured data. Natural language processing (NLP) offers a novel perspective for AE detection in free text versus relying on manual reading and coding of records for data collection. Young et al. recently conducted a systematic review of AE detection in incident reporting, EMRs, and morbidity and mortality records using NLP [7]. Of 35 papers, 9 used EMR data. Murff et al. developed free text searching of postoperative occurrences/complications (i.e., acute renal failure requiring dialysis, deep vein thrombosis, pulmonary embolism, sepsis, pneumonia, or myocardial infarction) in EMRs at six Veterans Health Administration medical centers [8]. The terms and descriptions were developed based on medical concepts in the Systematized Nomenclature of Medicine—Clinical Terms. The NLP algorithms were validated in 2974 charts that nurses reviewed. Sensitivity was lowest for deep vein thrombosis/ pulmonary embolism (59%), and highest for myocardial infarction (91%) among the complications studied. FitzHenry and Murff et al. overcame the major weakness of their previous study by establishing a training database [9]. They conducted chart reviews (7,743 patients) and split about 50% of reviews as training data and the remaining 50% as testing data. The study reported a wide range of sensitivities (ranging: 56–95%) and relatively high specificities (ranging 80–94%) for nine adverse events but low PPV for nine adverse events (ranging: 15% for deep vein thrombosis to 58% for pneumonia). Toyabe and colleagues reported that the performance of an EMR-based algorithm to identify inpatient falls varied in different sources of EMRs [10]. Although sensitivity was high (i.e., 100%), PPV was only 6% for progress notes, 13% for discharge summaries, and 100% for incident reports and image order entries. Searching entire EMRs would improve the performance of fall detection.

Current literature suggested that only a limited number of EMR algorithms have been developed for limited categories of AEs, and there are no systematic efforts for validation. ***The primary objectives*** of this project are, 1) to develop algorithms that have great potential to be used nationally or internationally to reduce the incidence of AEs through accurate and timely AE detection, and 2) to validate the developed EMR-based AE algorithms using chart review as the reference standard.

## Methods

### Study design and settings

**Fig 1:**
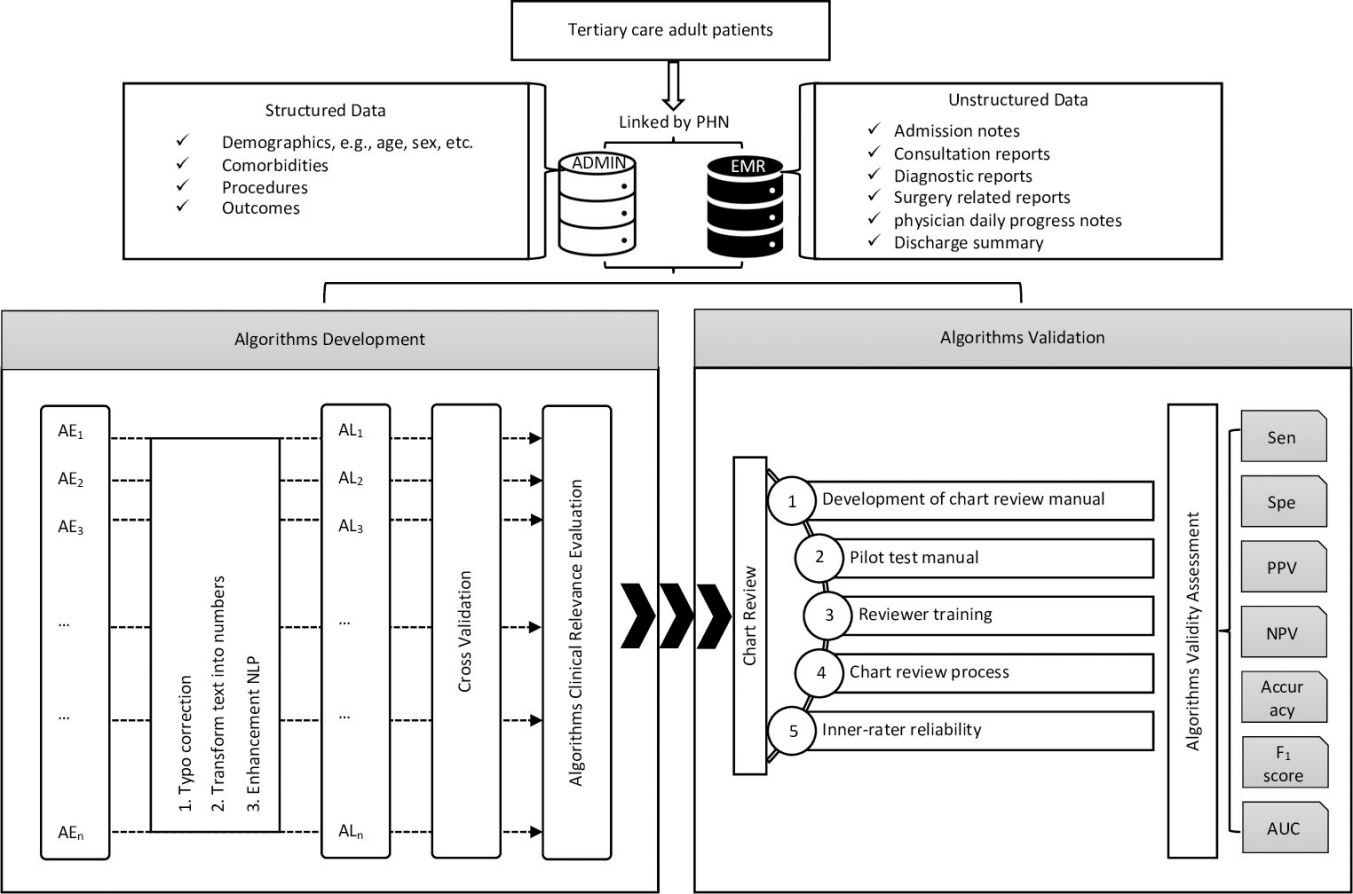
Research flow diagram. Abbreviations: ADMIN, administrative data, AE, adverse event, AL, algorithm, AUC, area under the receiver operating characteristic curve, EMR, electronic medical record, NPV, negative predictive value, PHN, personal health number, PPV, positive predictive value, Sen, sensitivity, Spe, specificity.

This retrospective, observational, multicentric cohort study will be conducted at four tertiary care hospitals in Calgary, Alberta, Canada (Fig 1).

### Population

We will randomly select patients who were 18 years or older and admitted to any acute care hospitals in Calgary, Alberta, from 2010 to 2020. If patients had multiple hospitalizations in this period on the most recent records will be included. Patients under 18 years old or over than 100 years old, and patients who did not have discharge summary records will be excluded from this study.

### Data collection

Data sources will include electronic medical records, which contain both structured field information (e.g., drop-down menus, numerical fields, checkboxes, radio buttons) and unstructured free-text data (e.g., discharge summaries, multidisciplinary progress notes, clinical examination, etc.). Discharge Abstract Data (DAD) contains demographic, abstracted administrative and clinical data on all hospital separations from acute inpatient facilities in Alberta. Each hospital discharge record includes up to 20 procedure codes and 25 diagnosis codes, recorded using the ICD-10-CA/CCI coding system. Two databases will be linked using personal health number and deidentified within Alberta Health Services prior to analysis.

### Algorithm development

Step 1: To generate adverse event list. Our comprehensive literature review identified a preliminary list containing 38 categories of AEs (Table 1).

**Table 1:** The adverse events list.

Calgary Adverse Events	
1.	Anesthesia Related Complications
2.	Cardiac Complications
3.	Central Nervous System Complications
4.	Delirium
5.	Drug Related Adverse Events
6.	Direct Surgery Complications
7.	Decubitus Ulcer
8.	Endocrine & Metabolic Complications (Electrolyte abnormalities, diabetes, etc.)
9.	Fluid Management
10.	Gastrointestinal
11.	Hospital Acquired Infection
12.	Hemorrhagic Events
13.	Obstetric Complications Affecting Fetus
14.	Obstetric Complications Affecting Mother
15.	Respiratory Complications
16.	Severe Life or Major Vital Organ Threatening Adverse Event
17.	Traumatic Injuries
18.	Venous Thromboembolic Events
AHRQ Adverse Events	
1.	Complications of anesthesia
2.	Death in low mortality DRGs
3.	Decubitus ulcer
4.	Failure to rescue
5.	Foreign body left in during procedure
6.	Iatrogenic pneumothorax
7.	Selected infections due to medical care
8.	Postoperative hip fracture
9.	Postoperative hemorrhage or hematoma
10.	Postoperative physiologic and metabolic derangements
11.	Postoperative respiratory failure
12.	Postoperative pulmonary embolism or deep vein thrombosis
13.	Postoperative sepsis
14.	Postoperative wound dehiscence in abdominopelvic surgical patients
15.	Accidental puncture and laceration
16.	Transfusion reaction
17.	Birth trauma, injury to neonate
18.	Obstetric trauma—vaginal delivery with instrumentation
19.	Obstetric trauma—vaginal delivery without instrumentation
20.	Obstetric trauma—cesarean delivery

Calgary Adverse Event List: Our team developed a comprehensive list of AEs [11]. First, we queried all acute care hospitalizations for a 1-year period in the national DAD for ICD-10-CA diagnosis codes that were coded as arising after admission to hospital (n = 2,590). Second, we undertook a modified Delphi panel process involving international clinicians with expertise in safety and quality to rate the extent to which each of the identified diagnoses has a potential link to suboptimal safety and quality of care. The panel selected 650 diagnosis codes as AEs. Third, we grouped these events into 18 relevant clinical categories.

AHRQ List: AHRQ PSIs were developed through a literature search, review of ICD-9-CM manuals, consultation with physician panels, and empirical data analyses. Over 200 ICD-9-CM codes representing potential patient safety problems were identified, and 48 indicators were labeled as the most promising PSIs by the AHRQ research team. Of these, 20 hospital-level PSIs were recommended by multi-specialty panels as a set of ‘accepted’ indicators for application in ICD administrative data [2, 3].

Step 2: To develop EMR-based algorithms by mapping AEs to EMR free texts through NLP. First, we will develop an algorithm to do typo corrections. As a proof of concept, based on a pre-trained network using medical terms and Aspell [12], an established spell checker, we developed a typo-corrector tailored for medical use. The typo correction algorithm is based on the distance between words. The distance between words means how many operations (deletion, insertion, or substitution) are needed to convert one to another. All words in the EMR that cannot be matched with the standard lexicon will be replaced with the existing words with the most similar medical terms. For instance, “urinalyses” is considered a typo because it is out of the prepared lexicon. Therefore, the computer will suggest correcting it with the word “urinalysis” as it needs only one substitution to convert the typo “urinalyses” to “urinalysis.” In order to adapt to EMR, 90,142 medical-related words have been added to the standard lexicon. Medical vocabulary comes from the two largest open-source libraries [13, 14], containing most medical-related words, including physiology, drugs, and disease names. We used this tool in a publicly available U.S. EMR (i.e., MIMIC-III) and manually inspected the corrected cases. The result showed that it corrected the misspelling and did not change the medical acronyms mistakenly. Specifically, an average of 22.5 words (2.37%) were corrected in one discharge summary. The same technique will be used with our EMR data.

Second, to transform text into analyzable numbers, we will combine the traditional Dataless Text Classification and the state-of-the-art pre-trained document-embeddings [15, 16]. In contrast to a standard supervised learning which needs human elaborated labels (i.e., gold standard), Dataless Text Classification uses the occurrence of keywords to label documents and thus is called semi-supervised learning. Dataless Text Classification initially assigns labels by investigating the existence of predefined keywords and then repeat assigning labels and model training. For example, to identify pressure ulcers, we can use a predefined list of descriptions, including pressure ulcer, skin breakdown from pressure, decubitus ulcers, bedsore, to give the initial labels to documents. The initial label will guide the first model, and then we update labels according to the prediction result made by the first model. This process will be repeated until the classification results cannot be improved. Thus, a well-defined and complete keyword list is crucial for the model’s effectiveness. Experts may develop a comprehensive list of keywords for one or several AEs; however, generating keyword lists for all AEs is unrealistic due to the large number of variant terms used in clinical notes. For example, frequent acronyms in EMRs such as “decub” for “decubitus” that is not known as a priori. Hence, we will use “word embedding,” enabling computers to automatically find more keywords relying on a few experts’ keywords. Word embedding is a technique where words or phrases are converted to vectors of real numbers, preserving their identity and relationship with others (i.e., context and semantics).

**Fig 2:**
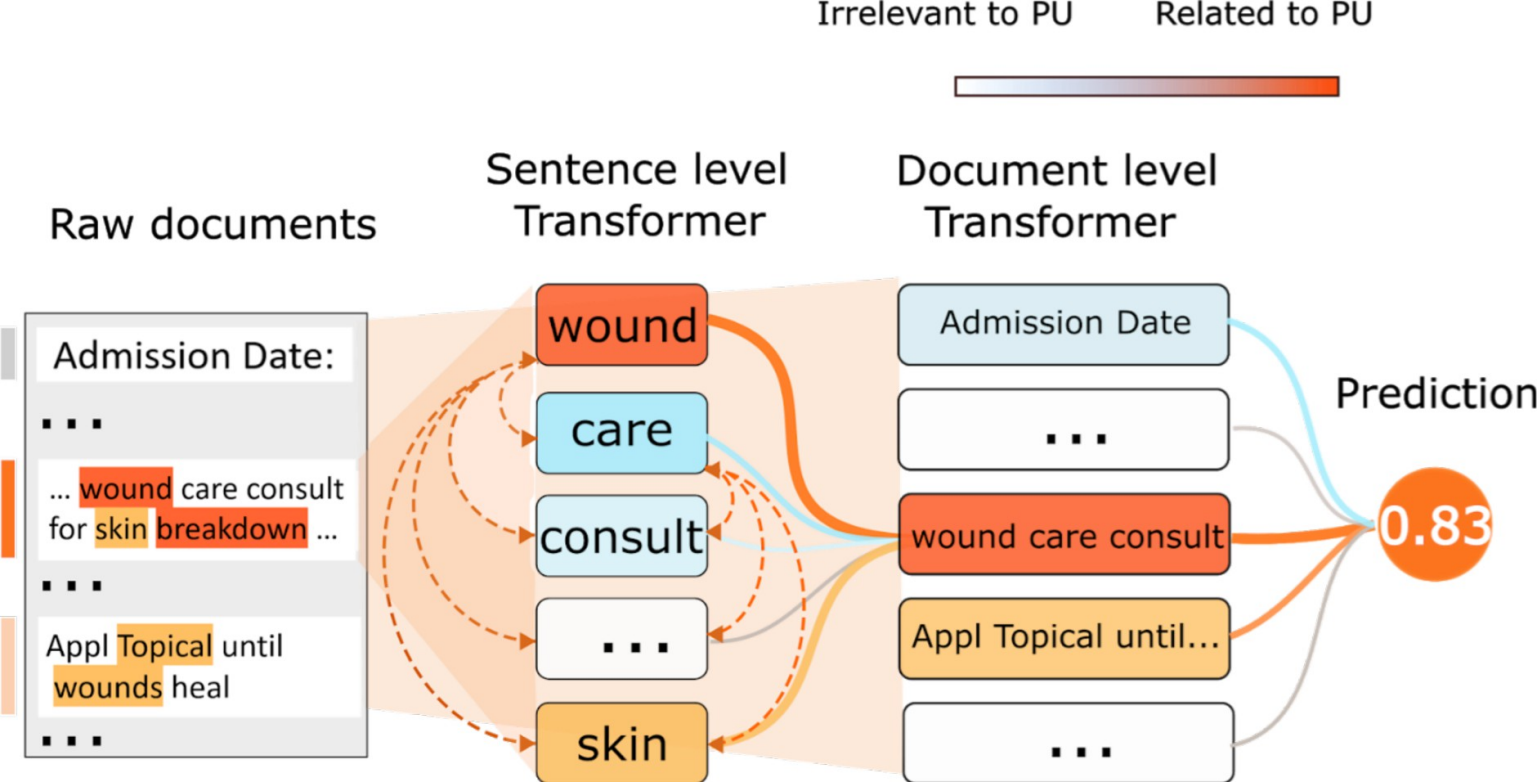
The process of text classification for pressure ulcers (PU). This picture shows the steps of text classification. We divide raw documents into sentences. All the words of each sentence are passed to the transformer to obtain a vector that can carry the sentence’s meaning. The vectors of all sentences are passed to another transformer to get the document embedding. The transformer can be seen as a "summarizer," step by step, the transformer abstract the meaning of the article related to the classification problem into the final document embedding.

The vector obtained by word embedding is usually sensible in terms of a word’s semantic meaning. At the same time, we need a model to process the transformed numbers, and the parameters of the model will be adjusted according to the context (surrounding words) of each word. The process of obtaining this model is called “Pretraining.” Intuitively, word embedding will explore more keywords because the potential keywords with similar meanings to the expert-defined keywords will be surrounded by similar context. We will use a state-of-the-art pretraining framework BERT (Bidirectional Encoder Representations from Transformers). We have implemented a prototype using BERT for keyword searching (Fig 2). By using BERT-based pretraining, the model can quickly learn that the word “decub” means the same as “decubitus.” Because a large number of words and interactions are involved in the pretraining process, the BERT model can generate a vector that can represent the entire sentence while giving each word a vector. This vector is called sentence embedding. Applying another BERT model to all sentences in a document can condense the meaning of the entire text into a vector; this vector is called document embedding. Further, training any classifier based on this document embedding will result in a document classification tool.

Third, to enhance the algorithm, we will use the DAD as the “reference standard” to identify AEs for guiding our NLP algorithm development. We will define 38 categories of AE in the DAD using the existing ICD-10 algorithms. However, the DAD is likely underreporting AEs [11]; thus, we do not use the DAD data as a “gold standard” in supervised training. Instead, we use semi-supervised learning integrating DAD as a reference standard, that means both DAD and predefined list of keywords will be exploited to produce the initial labels. AEs without a label can be well handled in the above “Second” step, and the ICD codes used to map AEs in DAD can contribute to better identifying AEs from free text. The semi-supervised learning method will align reference standard data and NLP-generated labels iteratively. In each round, we will retrain the model using BERT-enabled NLP algorithms to generate labels for all unlabeled charts with confidence scores. Highly confident labels will be noted separately as a “silver-standard” subgroup. By iteratively retraining the model, the NLP method will correct the under-reporting of AEs in DAD, leading to high sensitivity. We have implemented a prototype of this method and tested it with MIMIC-III data, and the preliminary result suggests that the “silver standard” labels are reliable (data not shown).

We estimated the appropriateness of the proposed approach by estimating the prevalence of AEs. Based on the previous analysis of DAD data, our team reported that the least prevalent AE is obstetrical complications affecting the fetus with 0.03% [11]. Therefore, our study population of over 1.5 million will result in a sample size of 450 for the least prevalent AE. Based on our experience with MIMIC-III data, this sample size is sufficient to guide models to identify AE cases.

Step 3: To assess the clinical relevance of the developed AE algorithms. To ensure the clinical relevance of the algorithm contents, we will form a panel of 9 experts consisting of physicians, nurses, patients, and clinical coding specialists) to review the information. They will independently review the algorithms and subsequently meet to discuss whether the results generated by each algorithm meet the clinical definition for the AE. Modified Delphi method will be employed to achieve consensus [17].

### Algorithm validation

All developed algorithms will be validated through a comprehensive extensive chart review (n = 10,000). We will first develop a practical manual to guide the chart review process. The presence of AE (AE definition, inclusion and exclusion criteria) and its related background information (e.g., timing of AE, severity classification, etc.) will be extracted by a secure web-based instrument called REDCap. Second, two clinical experts will conduct a pilot chart review of 50 charts to revise and prioritize the manual. Third, all chart reviewers will undergo pre-designed training. We will test 25 charts for data extraction. Discrepancies raised from this session will be discussed and resolved by consensus. A third party of experts will be involved to reach a consensus. The reviewers will then review another 25 charts independently to assess the consensus for each AE category. The agreement between the reviewers in each AE category will be examined. If the agreement is not substantial (kappa<60%), further training will be provided, and an additional 25 charts will be reviewed to retest the agreement. Fourth, to conduct the chart review, the reviewers will independently extract data from the entire chart, including the cover page, discharge summaries, narrative summaries, pathology reports (including autopsy reports), trauma and resuscitation records, admission notes, consultation reports, diagnostic reports, surgery reports, anesthesia reports, and physician daily progress notes for evidence of AEs. Reviewers will follow the AE definitions provided in the manual to determine the presence or absence of AEs and specify whether the AE was present at the time of admission or arose during hospitalization. Last, various statistical analyses will be used to thoroughly assess the AE algorithms’ properties. We will calculate standard statistics, including sensitivity, specificity, positive predictive value, negative predictive value (NPV), F1-score, as well as AUC (area under the receiver operating characteristic curve) to assess the algorithms accepting chart review results as the reference standard.

### Sample size

The estimated sample size of chart review is 10,000. We aim to have algorithms with high sensitivities and PPVs, given that specificity and NPV are likely very high (>95%) due to rare events. To confidently calculate these validity measures, we aim to have at least 10 positive cases for each AE category. To obtain 10 positive cases for those AEs with higher than 0.1% prevalence, we need to review 10,000 charts. Of included AE categories, DAD analysis shows that three AEs have a prevalence lower than 0.1%, including endocrine and metabolic complications (0.09%), obstetrical complications affecting the fetus (0.03%) and anesthesia-related complications (0.04%) [11]. However, the prevalence is likely underestimated [18, 19]; our chart review should have a higher prevalence than these reports.

## Discussion

This present study protocol described a methodological framework of developing machine learning algorithms based on structured and unstructured EMR data to automate AEs surveillance and promote healthcare system performance. We will validate developed algorithms through a large cohort of chart review to ensure their applicability and generalizability. The results of this project will improve the AE case identification and create a pathway to robust and valid prediction models in healthcare system [20, 21].

The strength of this proposed project includes: first, the AE algorithms will be developed based on a large scale of EMR data sampled from 1.5 million individual patients and further validated with over 10,000 medical expert chart review data. Second, to increase the generalizability of study results nationally and internationally, we will only include common EMR contents for algorithms development, such as discharge summaries, nurse notes, diagnostics reports and surgery notes which are key and commonly standard contents among different hospital EMRs. Third, the methodological framework we developed in this study can be applied to other initiatives that attempt to leverage EMR data for enhanced AE detection, given the broad implementation of EMRs internationally.

A few limitations need to be considered in this project. First, AE algorithms derived from this study will be developed from EMR data of tertiary care hospitals. Its generalizability to rural care is unknown. Second, algorithms will be developed with a local EMR database named SCM. We acknowledge that the structure of EMR may differ from one care territory to another. However, key features of EMR are similarly collected across healthcare systems. When applying our algorithms, end-users need to match the content we used to the corresponding contents in their EMR. Our final AE algorithms will contain a detailed description of what content is used in the algorithm and the data format. Third, institutional factors (e.g., culture, policies, etc.) may influence how AEs are documented in EMR. We conducted a literature review on interventions to improve EMR documentation [22]. Clinician education stood out as a primary intervention. The major challenge is quantifying the quality of EMR documentation and measuring the effectiveness of interventions. To partially address this issue, we will conduct a stratified analysis to assess the incidence of AEs in EMR algorithms in different hospitals, units, or disciplines. However, the completeness of documentation of AEs in EMR is at another level of detail and out of the scope of this proposed project and deserves a separate study. While some institutional factors may affect the recorded incidence of a specific AE in EMR to some degree, this should not be a barrier to the application of our NLP method to other EMRs.

## List of abbreviations

AE: adverse event
AHRQ: Agency for Healthcare Research and Quality
AUC: area under the receiver operating characteristic curve
DAD: discharge abstract data
EMR: electronic medical records
NLP: Natural Language Processing
NPV: negative predictive value
PPV: positive predictive value
PSIs: Patient Safety Indicators
SCM: Sunrise Clinical Manager
